# In Vitro and In Vivo Investigations into the Potential of Quinazoline and Quinoline Derivatives as NorA Efflux Pump Inhibitors Against Resistant *Staphylococcus aureus* Strains

**DOI:** 10.3390/antibiotics14040339

**Published:** 2025-03-26

**Authors:** Nishtha Chandal, Nidhi Sharma, Giada Cernicchi, Tommaso Felicetti, Tommaso Rondini, Mattia Acito, Hemraj Nandanwar, Stefano Sabatini

**Affiliations:** 1 Clinical Microbiology & Antimicrobial Research Laboratory, CSIR—Institute of Microbial Technology, Sector 39-A, Chandigarh 60036, Punjab and Haryana, India; nishthachandal@gmail.com (N.C.); sharmanidhi@imtech.res.in (N.S.); 2 Academy of Scientific & Innovative Research (AcSIR), Ghaziabad 201002, Uttar Pradesh, India; 3 Institute of Biosciences and Technology, Texas A & M Health Science Center, Houston, TX 77807, USA; 4 Department of Pharmaceutical Sciences, Section of Chemistry and Drug Technology, University of Perugia, 06123 Perugia, Italy; giada.cernicchi@unipg.it (G.C.); tommaso.felicetti@unipg.it (T.F.); 5 Department of Pharmaceutical Sciences, Section of Biochemical and Health Sciences, University of Perugia, 06123 Perugia, Italy; tommaso.rondini@dottorandi.unipg.it (T.R.); mattia.acito@unicam.it (M.A.); 6 School of Medicinal and Health Products Sciences, University of Camerino, 62032 Camerino, Italy

**Keywords:** efflux pump inhibitors (EPIs), *Staphylococcus aureus*, antimicrobial resistance (AMR), antimicrobial resistance breakers (ARBs), ciprofloxacin

## Abstract

**Background:** *Staphylococcus aureus* is a highly lethal Gram-positive bacterium that is responsible for over one million deaths annually. As a member of the ESKAPE pathogens, its methicillin-resistant strains (MRSA) are prevalent worldwide and exhibit significant antimicrobial resistance (AMR). Bacterial efflux pumps play a pivotal role in the development of AMR by facilitating the expulsion of a range of antimicrobial agents. **Methods**: The *S. aureus* strain SA-1199B, which overexpresses NorA and carries a GrlA mutation, was utilized to comprehensively profile the mechanism of the compounds **PQQ16P** and **PQK4F**. To assess the toxicity and genotoxicity of these compounds, RAW macrophages, HEK 293T, and HepG2 cell lines were utilized. Female BALB/c mice were utilized to assess the in vivo synergism of EPIs with CPX, **Results**: NorA efflux pump inhibitors (EPIs), **PQQ16P** and **PQK4F**, enhanced the efficacy of the antibacterial ciprofloxacin (CPX) against resistant *S. aureus* strains. The mechanism of EPIs involved the inhibition of NorA efflux pump, without compromising bacterial membrane permeability, ATP levels, or mammalian calcium channels. Moreover, the EPIs significantly augmented the bactericidal and post-antibiotic effects of CPX, elevating its mutation prevention concentration without manifesting substantial toxicity to human cells. Furthermore, the EPIs reduced *S. aureus* invasiveness in macrophages, indicating a role for NorA in bacterial virulence. Notably, the in vivo synergism of these EPIs with CPX was observed in a mouse infection model. **Conclusions**: This study provides substantial evidence for the potential of employing EPIs in a combination with CPX to counteract AMR, both in vitro and in vivo.

## 1. Introduction

*Staphylococcus aureus* is a Gram-positive bacterial pathogen that is a leading cause of both hospital and community-acquired infections [1]. Due to its capacity for adaptation to diverse environmental conditions, *S. aureus* is capable of causing a broad spectrum of infections, ranging from minor dermatological conditions to severe diseases such as pneumonia, endocarditis, and sepsis [2,3,4]. The pathogenicity of *S. aureus* is largely attributed to a diverse array of virulence factors, including toxins and enzymes that facilitate tissue invasion and immune evasion [2]. *S. aureus* is one of the most lethal bacterial pathogens, responsible for approximately 1.1 million deaths globally in 2019 [5]. In addition, it plays a significant role in the development of antimicrobial resistance (AMR) due to its capacity to evolve resistance to a range of antibiotics [6,7,8]. It is, therefore, unsurprising that it is part of the ESKAPE pathogens [9,10]. One of the most notable examples is methicillin-resistant *S. aureus* (MRSA), which exhibits resistance to methicillin and other beta-lactam antibiotics [11]. The treatment of MRSA infections presents a significant challenge, with higher morbidity and mortality rates associated with these infections [11]. The extensive utilization of antibiotics in both healthcare and agricultural contexts has facilitated the emergence of antibiotic-resistant strains of *S. aureus* [12,13,14]. This bacterium can obtain resistance genes through horizontal gene transfer and mutations, rendering it a formidable pathogen in both hospital and community settings [15]. The prevalence of MRSA and other resistant strains of *S. aureus* highlights the urgent necessity for the development of novel antibiotics and alternative therapeutic strategies to combat these infections [16]. Efflux pumps are of pivotal importance with regard to the antibiotic resistance of *S. aureus* [17,18]. These pumps are integral membrane proteins that actively expel a variety of antimicrobial agents out of the bacterial cell, thereby reducing the intracellular concentration of these drugs and diminishing their efficacy. In *S. aureus*, several efflux pumps have been identified, including NorA, NorB, NorC, MepA, and QacA/B [19,20,21]. These pumps are capable of transporting a diverse range of antibacterials, including macrolides, quinolones, and tetracyclines, as well as biocides such as quaternary ammonium compounds [19,20,21]. As a result, they play a pivotal role in enabling the bacterium to withstand the presence of these antimicrobial agents. Efflux pumps are frequently encoded on plasmids or within the bacterial chromosome, thereby facilitating the rapid acquisition and dissemination of resistance traits [22]. This renders them a pivotal element in the emergence of multidrug-resistant (MDR) and extensively drug-resistant (XDR) variants of *S. aureus*. In this regard, a challenge strategy is based on the approach of targeting and disrupting the mechanisms responsible for AMR. The intriguing concept of freezing resistance could potentially salvage ineffective antibiotics, thereby replenishing our arsenal of antimicrobial agents. The molecules that have been identified as capable of counteracting AMR have been referred to by a number of different names, including adjuvant molecules, helper compounds, and antimicrobial resistance breakers (ARBs) [23,24,25,26,27]. All are distinguished by a lack of antimicrobial activity and the capacity to interact synergistically with known antimicrobials, thereby restoring their efficacy against resistant strains. It is well established that microorganisms typically develop resistance only to substances that kill or inhibit their growth [28,29]. Consequently, the absence of direct antimicrobial activity in ARBs may prove advantageous for their future applications. Given the central role of bacterial efflux pumps in AMR, the development of efflux pump inhibitors (EPIs) is a valuable approach [30].

NorA, a transmembrane protein belonging to the Major Facilitator Superfamily (MFS) that utilizes the proton motive force (PMF) for energy, is the most extensively studied efflux pump in *S. aureus*. It has been observed to expel a variety of compounds (including the fluoroquinolone ciprofloxacin (CPX) and the dye ethidium bromide (EtBr)) [31,32,33]. Over time, researchers have identified numerous NorA EPIs through the application of three distinct methodologies: (i) screening of compound libraries, both natural and synthetic, (ii) the repurposing of existing drugs, and (iii) the design and synthesis of new compounds based on observed effects [34]. However, only the recent release of two three-dimensional structures of NorA [33,35] and the lack of biophysical/biochemical assays using the isolated protein have significantly impeded structure-based drug design and the identification of potent NorA EPIs, which have yet to reach clinical trials. Nevertheless, some chemical classes of NorA EPIs have been identified, including indoles [36,37,38,39], quinolines [40,41], quinazolines [42], boronic acids [43,44], chalcones [45], and piperine derivatives [46,47], as well as also various natural compounds. These include boeravinone B, capsaicin, α-terpinene, eugenol, isoeugenol, sesquiterpenes derived from *Pilgerodendron uviferum*, and some flavonoids that have demonstrated promising results [48,49,50,51,52,53]. Furthermore, plant extracts or essential oils, including the ethanolic extract from *Bauhinia forficata* leaves, *Chenopodium ambrosioides* L. essential oil, and *Nigella sativa* essential oil, have demonstrated promising outcomes [54,55,56].

**Figure 1:**
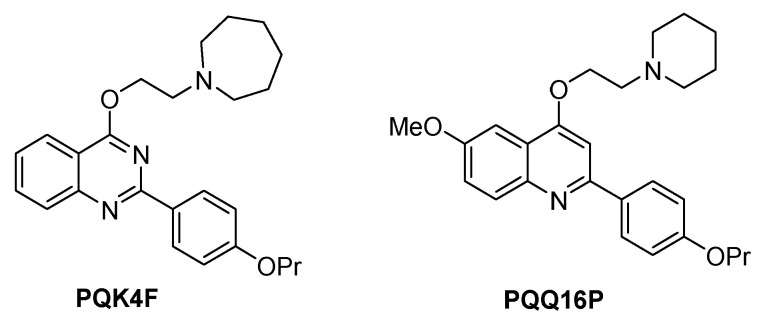
Chemical structures of NorA EPIs **PQK4F** and **PQQ16P**.

Our research group has been engaged in this field for years and has contributed to the development of various NorA EPIs through the application of different methodologies [57]. As a result of these endeavors, we recently reported the optimization of two classes of NorA EPIs derivatives: quinazolines and quinolines, from which the compounds **PQK4F** [42] and **PQQ16P** [58] emerged (Figure 1). Both demonstrated potent NorA inhibitory activity and a high capacity to synergize with CPX against resistant strains of *S. aureus*. In view of these encouraging outcomes, the present study was undertaken to conduct comprehensive investigations into the mechanisms of action of these two derivatives and their potential for combination with CPX, both in in vitro studies and in animal models.

## 2. Results

### 2.1. Impact of Compounds **PQK4F** and **PQQ16P** on CPX’s Minimum Inhibitory Concentration (MIC)

Firstly, checkerboard assays of **PQK4F** [42] and **PQQ16P** [58] in combination with CPX were carried out in order to confirm previous data against the resistant *S. aureus* strain SA-1199B (overexpressing *norA* and bearing a GrlA mutation on the fluoroquinolone target) [59] (Table S1). Additionally, determination of MIC values of **PQK4F** and **PQQ16P** was also evaluated to confirm the absence of any antibacterial effect at low concentrations when tested individually (Table S1). Indeed, as anticipated in the Introduction, EPIs are not expected to have antibacterial activity on their own. As expected, both compounds showed a promising synergism with CPX, reducing its MIC by 4-fold when tested at 3.13 µg/mL. To further confirm the synergism, Fractional Inhibitory Concentration Index (FICI) values were calculated and were ≤0.5 for both compounds, indicating a synergistic interaction with CPX (Table S1). In parallel, through checkerboard assays against *S. aureus* K-1758 (*norA* deletion strain), minimal or no modulation of the MIC values of CPX was observed for both compounds, as confirmed by FICI values > 0.5 to <4, suggesting an indifferent interaction (Table S2). Therefore, the observed synergy with CPX against *S. aureus* SA-1199B, which overexpresses the *norA* gene, suggests that the compounds **PQK4F** and **PQQ16P** may function as inhibitors of the NorA efflux pump, and this is further corroborated by the absence of synergism against the *S. aureus* K-1758 strain that does not express the *norA* gene.

### 2.2. Effect of the Combination of CPX with Compounds **PQK4F** and **PQQ16P** on the Time–Kill Kinetics of S. aureus SA-1199B

**Figure 2:**
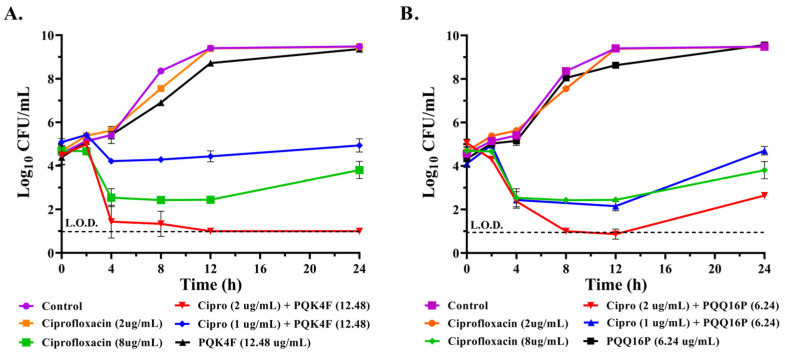
Time–kill kinetic assay of the combination of CPX (2 and 1 µg/mL) and **PQK4F** (**A**) and **PQQ16P** (**B**) at 12.48 and 6.24 µg/mL, respectively, on *S. aureus* SA-1199B strain. Control refers to the drug-free control (negative control), while CPX at its MIC (8 µg/mL) represents the positive control. The experiment is the representation of three individual repeats ± SD.

The time–kill kinetic study establishes the rate at which a compound kills a microorganism as a function of survival recorded at various exposure time points. Initially, CPX was used in this assay at inhibitory (8 µg/mL) and sub-inhibitory (2 µg/mL) concentrations alone. CPX at 8 µg/mL showed a reduction in SA-1199B growth of 2.25 log_10_ after 12 h of treatment, followed by a resurgence in growth. Conversely, CPX at 2 µg/mL showed a growth curve comparable to that of the untreated control bacterial cells. As expected, compounds **PQK4F** and **PQQ16P** tested individually at 12.48 µg/mL and 6.24 µg/mL, respectively, also demonstrated a comparable trend with no discernible impact on *S. aureus* growth. It is noteworthy that when sub-inhibitory concentrations of CPX (2 µg/mL and 1 µg/mL) were combined with **PQK4F** (12.48 µg/mL) or **PQQ16P** (6.24 µg/mL), a reduction of 3.69 log_10_ and 0.8 log_10_ and of 3.83 log_10_ and 2.54 log_10_, respectively, was observed after 12 h of treatment (Figure 2). Taken together, the data clearly demonstrate the significant impact of a combination of these two EPIs with CPX on the growth rate of the resistant *S. aureus* SA-1199B over time, thereby underscoring the potential benefits of a combination therapy.

### 2.3. Membrane Permeabilization and Depolarization Activity of Compounds **PQK4F** and **PQQ16P** on S. aureus SA-1199B

**Figure 3:**
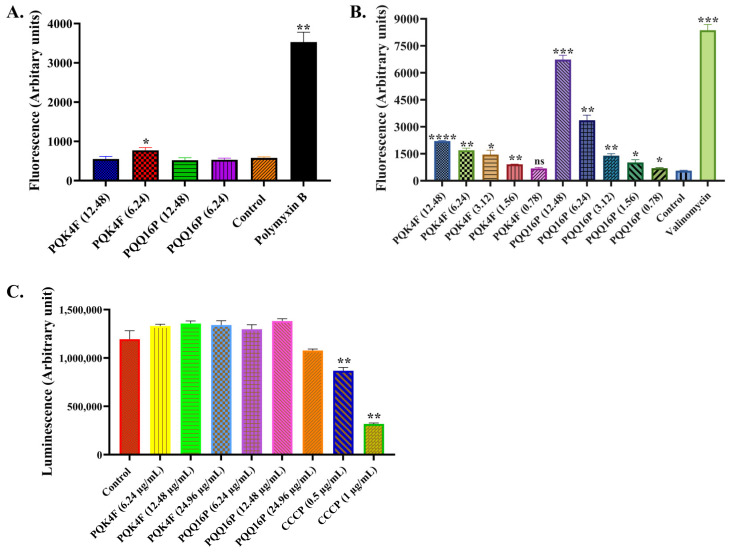
(**A**) Membrane permeability was measured using nucleic acid binding dye propidium iodide, polymyxin B was used as the positive control, and drug-free control was used as the negative control. (**B**) Membrane depolarization was detected using fluorescence of DiSC_3_(5) showing high fluorescence of positive control valinomycin; drug-free control was used as the negative control. (**C**) ATP bioluminescence detection assay. CCCP, the positive control, quenches ATP compared to control. All the results are representation of three individual readings ± SD on SA-1199B. Two-tailed *t*-tests were conducted to detect group variances. Statistical significance was defined as * *p* < 0.05, ** *p* < 0.01, *** *p* < 0.001, **** *p* < 0.0001. *p* value calculated using 95% class interval.

To exclude the possibility that the observed synergistic activity between CPX and **PQK4F**/**PQQ16P** was due to an unspecific effect generated by an increase in the *S. aureus* membrane permeability, the impact of **PQK4F** and **PQQ16P** on membrane permeabilization was assessed using propidium iodide (PI) (Figure 3A). Polymyxin, used as a positive control, exhibited a significant increase in the fluorescence. In contrast, no such increase was observed in the case of **PQK4F** and **PQQ16P** at concentrations of 6.24 and 12.48 µg/mL, respectively. This indicates that PI was unable to enter the viable cell and bind to the target. Taken together, the results demonstrated that the compounds **PQK4F** and **PQQ16P** do not permeabilize the membrane.

An additional phenomenon that could result in unspecific synergism and produce extended human cell toxicity is that of membrane depolarization. To assess whether compounds **PQK4F** and **PQQ16P** possessed this unwanted property, the membrane potential-sensitive dye DiSC_3_(5) was used to quantify changes in the electrical potential gradient in intact bacteria (Figure 3B). Valinomycin, used as a positive control, caused massive fluorescence leakage, demonstrating its membrane-depolarizing property [60]. Considering the two tested compounds, the effect was more pronounced for the compound **PQQ16P**, which at a concentration of 12.48 µg/mL led to a significant depolarization of the *S. aureus* membrane. However, at a concentration of 6.25 µg/mL, the membrane depolarization was observed to be relatively mild, and it nearly disappeared at a concentration of 3.13 µg/mL. These findings align with those of our previous research [58]. In contrast, the compound **PQK4F**, for which no previous evaluations of its impact on bacterial membrane depolarization have been conducted, demonstrated a markedly less pronounced effect than **PQQ16P**, even at the highest concentration (12.48 µg/mL). Given that NorA is a PMF-dependent pump, it is plausible that the compounds at the highest tested concentration (12.48 µg/mL) may disturb the PMF, thereby partially “freezing” the activity of the pump and contributing to the synergism with CPX through this “dirty” mechanism. Nevertheless, at lower concentrations (3.13 µg/mL and 6.25 µg/mL, particularly for **PQK4F**), the impact on membrane depolarization is insignificant, and the synergism of **PQK4F** and **PQQ16P** with CPX appears to be exclusively due to the direct inhibition of the NorA pump, rather than the disruption of the PMF (Figure 3B)**.**

### 2.4. Effect of Compounds **PQK4F** and **PQQ16P** on the Electron Transport Chain by ATP Bioluminescence Detection Assay

Membrane dysfunction disturbs the respiratory chain functions, resulting in reduced ATP levels. The *S. aureus* SA-1199B strain was incubated with **PQK4F** and **PQQ16P** at 6.24, 12.48, and 24.96 µg/mL for 5 h, and the effect of compounds on the intracellular ATP level was evaluated (Figure 3C). The levels of ATP remained constant in comparison to the drug-free control, thereby indicating that there has been no change in transmembrane potential. Consequently, it can be ruled out that ATP depletion was the cause of the efflux inhibition. Conversely, a substantial decline in the intracellular ATP was evident when using the positive control CCCP, a highly effective mitochondrial uncoupling agent that augments the proton permeability across the inner mitochondrial membranes [30].

### 2.5. Effect of Compounds **PQK4F** and **PQQ16P** on Mammalian Ca^2+^ Channels

**Figure 4:**
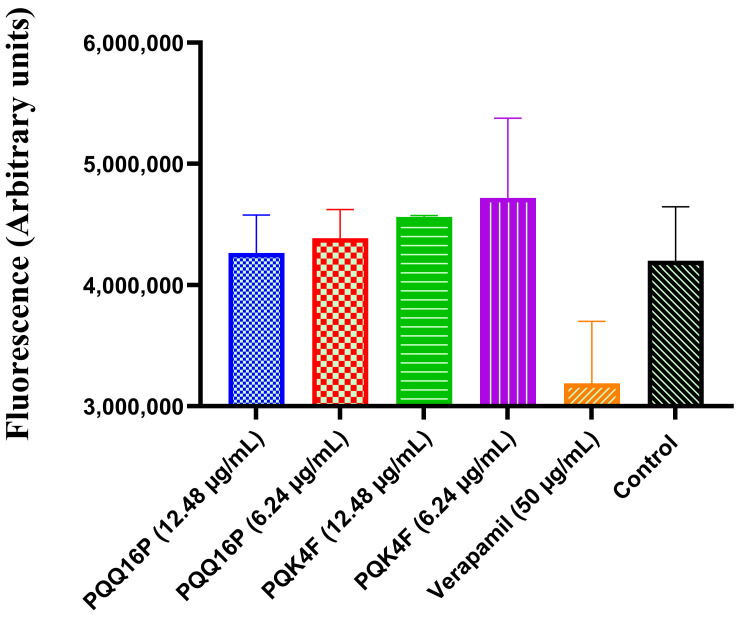
Activity of **PQK4F** and **PQQ16P** on mammalian Ca^2+^ channels of HEK cells. The results are the representation of three individual readings ± SD.

It is well established that bacterial efflux inhibitors frequently impose limitations on mammalian Ca^2+^ channel activity in clinical contexts. For example, verapamil has been observed to impede the function of human Ca^2+^ channels, which has been implicated in the manifestations of adverse neurological consequences in humans [61,62]. Accordingly, using HEK 293T cells and the Fluo-4 Direct calcium channel test kit, the blockage of the mammalian Ca^2+^ channel was evaluated (Figure 4). It is noteworthy that the activity of the Ca^2+^ channel was not affected by **PQK4F** and **PQQ16P** at concentrations of 12.48 and 6.24 µg/mL. In contrast, the positive control, verapamil, prevented the accumulation of Ca^2+^ ions in the cytoplasm. Taken together, the results showed that the compounds did not disturb the Ca^2+^ channel activity, overcoming the problem that has hampered the development of some EPIs [63,64] (Figure 4).

### 2.6. Toxicity Studies of Compounds **PQK4F** and **PQQ16P** Towards Eukaryotic Cells

**Figure 5:**
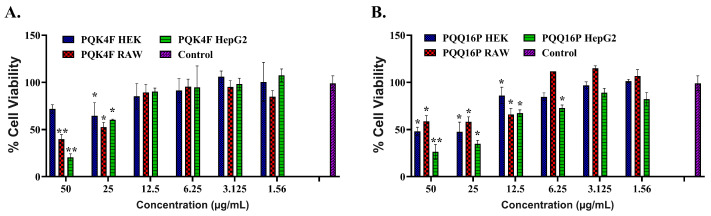
Cytotoxicity assay on human embryonic kidney cells (HEK 293T), RAW, and HepG2 cells of **PQK4F** (**A**) and **PQQ16P** (**B**) at concentrations ranging from 50 to 1.56 µg/mL. The results are the representation of three individual readings ± SD. Two-tailed *t*-tests were conducted to detect group variances. Statistical significance was defined as * *p* < 0.05, ** *p* < 0.01. *p* value calculated using 95% class interval.

In order to gain preliminary insight into the toxicity of compounds **PQK4F** and **PQQ16P**, a viability assessment of mammalian cells was conducted on three different cell lines: RAW macrophage cells, human embryonic kidney cells (HEK 293T), and hepatoblastoma cell line (HepG2). Cell viability was determined by MTT assay at varying concentrations of **PQK4F** and **PQQ16P** ranging from 1.56 to 50 µg/mL (Figure 5). In general, both compounds demonstrated a certain degree of toxicity at concentrations of 50 and 25 µg/mL, while at lower concentrations, they exhibited significantly reduced toxicity. It is noteworthy that a comparison of the profiles of **PQK4F** and **PQQ16P** revealed that the former exhibited a superior safety profile and was significantly safer than the quinoline derivative **PQQ16P**. However, when the focus is narrowed to the concentrations utilized to achieve a notable synergistic effect with CPX, it can be observed that both compounds do not exhibit a considerable degree of toxicity against the three cell lines. This suggests that the synergistic effect can be attained at concentrations that are not inherently toxic.

### 2.7. Hemolytic Activity Studies of the Compounds **PQK4F** and **PQQ16P**

**Figure 6:**
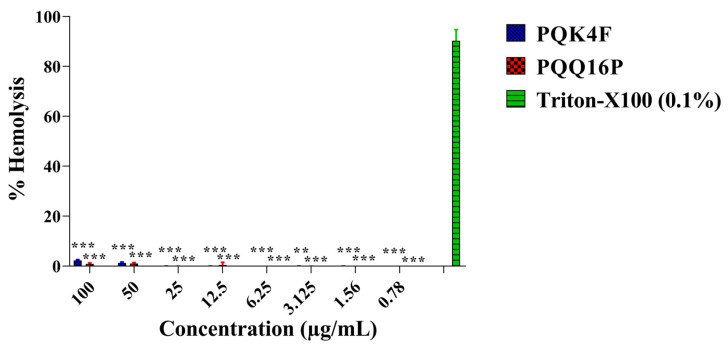
Hemolysis assay on 4% RBC at 100 to 0.78 µg/mL concentration. The results are the representation of three individual readings ± SD. Two-tailed *t*-tests were conducted to detect group variances. Statistical significance was defined ** *p* < 0.01, *** *p* < 0.001. *p* value calculated using 95% class interval.

Additionally, the hemolytic activity of both compounds was evaluated by measuring the release of hemoglobin from rabbit erythrocytes as a function of concentration. It is noteworthy that the compounds **PQK4F** and **PQQ16P** exhibited minimal hemolytic activity, with values of 2.2% and 0.8%, respectively, at a concentration of 100 µg/mL (Figure 6)**.**

### 2.8. Genotoxicity Assessment

**Figure 7:**
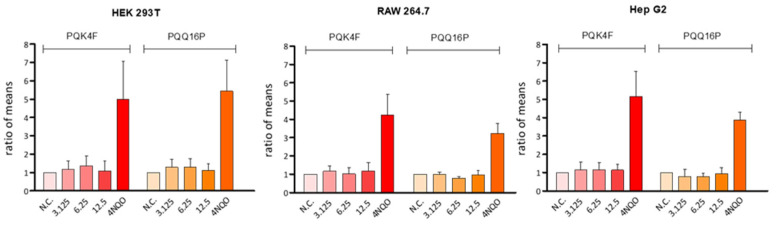
Genotoxic effects of **PQK4F** and **PQQ16P** antibiotics in HEK 293T, RAW 264.7, and HepG2 cells after 4 h of exposure. Each result is expressed as the ratio of means with a 95% confidence interval of three independent experiments. Negative control (N.C., untreated cells); positive control (2 μM 4NQO).

The assessment of genotoxicity in preclinical candidates is of paramount importance for the early identification of compounds that may induce genetic mutations, which can ultimately lead to the development of cancer or heritable genetic defects. Indeed, the early detection of genotoxicity in the drug development process can result in significant savings in terms of time and resources. Such an approach prevents the advancement of potentially harmful compounds into later stages of development, where the costs and time investments are significantly higher. Accordingly, comet assays were conducted to assess the potential genotoxicity of **PQK4F** and **PQQ16P** using three distinct cell lines. It is noteworthy that the two compounds demonstrated no genotoxic effect (Figure 7, Supplementary Table S3). The ratio of means for the cells exposed to the compounds ranged between 0.78 and 1.37, while the ratio of means for the positive control (2 µM 4NQO) fluctuated between 3.23 and 5.45.

### 2.9. Enhanced Post-Antibiotic Life of CPX in the Presence of Compounds **PQK4F** and **PQQ16P**

**Figure 8:**
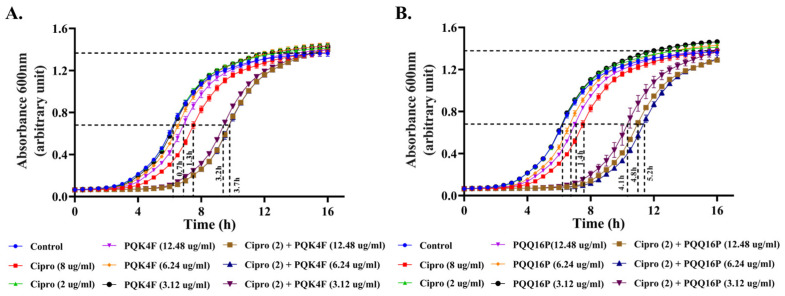
Post-antibiotic effect of CPX at 2 and 8 µg/mL alone and in combination with **PQK4F** (**A**) and **PQQ16P** (**B**) at 12.48, 6.24, and 3.12 µg/mL alone and in combination with CPX at 2 µg/mL by turbidity method on *S. aureus* SA-1199B strain in h; the results are the representation of three individual readings ± SD.

The post-antibiotic effect (PAE) is defined as the suppression of bacterial growth for a specified duration following a brief exposure to an antimicrobial agent. A longer PAE allows for the administration of antibiotics at less frequent intervals, which can enhance patient compliance and mitigate the risk of missed doses. Moreover, extending the PAE can guarantee that bacteria are subjected to the antibiotic’s effects for a more extended period, which may result in a more efficacious eradication of the infection. When an exponential-phase culture of *S. aureus* SA-1199B was subjected to 1 × MIC of CPX alone, a PAE of 1.3/1.4 h was observed. In contrast, a notable increase in the PAE was observed when CPX (2 µg/mL) was combined with **PQK4F** and **PQQ16P** at three distinct concentrations (12.48, 6.24, and 3.12 µg/mL). In particular, the increase in PAE in combination with **PQK4F** was of 3.7, 3.7, and 3.2 h, respectively, while in combination with **PQQ16P**, it was of 5.2, 4.8, and 4.1 h. The findings suggest that both EPIs contribute to an extension in the period of bacterial growth suppression (Figure 8).

### 2.10. Mutation Frequency Analysis of Compounds **PQK4F** and **PQQ16P** to Check the Development of Its Resistant Mutants

The capacity of a compound to impede or attenuate the emergence of resistant mutants is quantified by its mutation prevention concentration (MPC). It is imperative to maintain antibiotic concentrations above the MPC to prevent the development of resistance through single-step mutations. Indeed, the mutant selection window represents the range of antibiotic concentrations where resistant mutants are likely to emerge. A reduction in the MPC will result in a narrowing of the window, thereby reducing the probability of the emergence of resistant mutants. CPX alone displayed an MPC at 4 × MIC. In comparison, **PQK4F** at 12.48 and 6.24 µg/mL reduced the MPC of CPX by 8- and 4-fold, respectively, in the *S. aureus* SA-1199B strain (Table 1)**.** Similar results were obtained when testing **PQQ16P** at 6.24 and 3.12 µg/mL in combination with CPX (Table 1).

### 2.11. Effectiveness of **PQK4F** and **PQQ16P** in Mitigating Macrophage Invasion by norA Over-Expressing S. aureus

**Figure 9:**
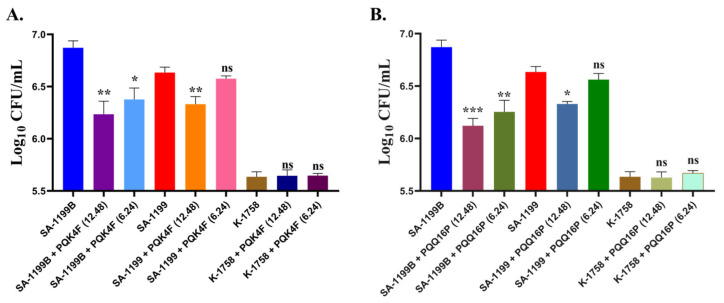
Macrophage invasion assays were conducted on *S. aureus* strains SA-1199B (*norA* over-expressing), SA-1199 (*norA* wild-type), and K-1758 (*norA* knockout) in the presence and absence of **PQK4F** (**A**) and **PQQ16P** (**B**) at 12.48 and 6.24 µg/mL concentrations; the results are the representation of three individual readings ± SD. Two-tailed *t*-tests were conducted to detect group variances. Statistical significance was defined as *p* < 0.05 (*), highly significant as * *p* < 0.05, ** *p* < 0.01, *** *p* < 0.001, ns = non-significant. *p* value calculated using 95% class interval.

It is of great importance to mitigate the invasion of macrophages by *S. aureus* for a number of reasons, including the prevention of chronic infections, reduction in immune evasion, limitation of tissue damage, enhancement of antibiotic efficacy, and the prevention of dissemination [65]. Accordingly, the intracellular invasion of macrophage THP-1 cells (3 × 10^5^ cells/well) by different *S. aureus* strains, including SA-1199B (*norA* overexpressing strain), SA-1199 (wild-type strain), and K-1758 (*norA* knockout strain), was evaluated in the presence or absence of **PQK4F** and **PQQ16P** (12.48 and 6.24 µg/mL) (Figure 9). The presence of **PQK4F** (12.48 and 6.24 µg/mL) led to a reduction in the invasion of *S. aureus* SA-1199B to 0.63 and 0.49 log_10_, respectively, compared to reductions of 0.30 and 0.05 log_10_, respectively, in the wild-type *S. aureus* SA-1199. Similarly, the presence of **PQQ16P** (12.48 and 6.24 µg/mL) resulted in decreased invasion of *S. aureus* SA-1199B to 0.75 and 0.61 log_10_, respectively, compared to reductions of 0.30 and 0.07 log_10_, respectively, in the wild-type SA-1199. The penetration of *S. aureus* K-1758 remained unaffected by **PQK4F** and **PQQ16P** treatments at concentrations of 12.48 and 6.24 µg/mL. Taken together, the data not only underscore the potential efficacy of the compounds **PQK4F** and **PQQ16P** in mitigating macrophage invasion by *S. aureus* but also indirectly reinforce the importance of efflux pumps in the invasion process. Indeed, it can be observed that the strain SA-1199B, which overexpresses *norA*, exhibits a markedly enhanced invasion capacity relative to both the wild-type strain and the strain that does not express *norA*. In particular, the difference in macrophage invasion capacity between the latter and the former is readily apparent. Moreover, these findings suggest that EPIs may serve as potential anti-virulence agents, given that when administered alone, they are capable of significantly reducing the macrophage invasion capacity of *S. aureus*.

### 2.12. Mouse Thigh Infection Model In Vivo

**Figure 10:**
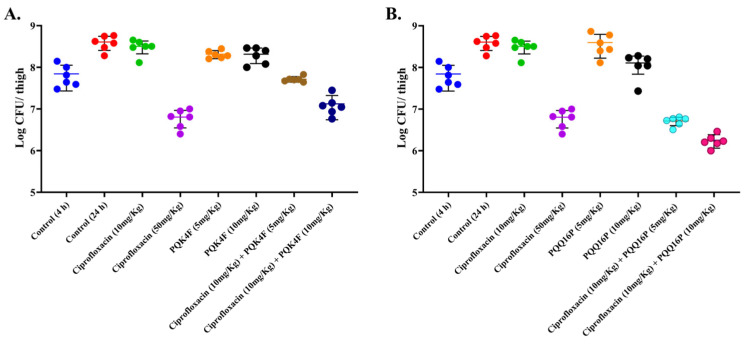
Neutropenic mouse thigh infection model. Single subcutaneous dose treatments of 5 and 10 mg/kg **PQK4F** (**A**) and **PQQ16P** (**B**), alone and in combination with 10 mg/kg CPX, administered 4 h post-infection, ciprofloxacin at 10 and 50 mg/kg was taken as positive control. Six mice per group were used. CFU determined 24 h post-infection for drug-treated mice; controls assessed at 4 and 24 h post-infection for CFU calculation.

The impressive efficacy of the combination prompted us to assess the impact of compounds **PQK4F** and **PQQ16P** in combination with CPX on a mouse soft-tissue infection model. Using a neutropenic mouse thigh infection model, we investigated the in vivo effectiveness of these compounds (Figure 10). Mice were infected intramuscularly with 10^7^ CFU of *S. aureus* SA-1199B. After 4 h, the first control group was sacrificed to confirm successful infection establishment, and their right thigh tissue was homogenized and plated. The CFU count in this control group was approximately 7.7 log_10_. Treatment with CPX (10 mg/kg) or the EPIs **PQK4F** and **PQQ16P** (5 and 10 mg/kg) alone, administered 4 h post-infection, did not significantly reduce bacterial counts after 20 h of treatment. However, combining **PQK4F** at doses of 5 mg/kg and 10 mg/kg with CPX at 10 mg/kg, administered 4 h post-infection, showed notable efficacy, resulting in a reduction of approximately 0.86 and 1.5 log_10_, respectively, compared to the untreated control after 24 h. Similarly, combining **PQQ16P** at doses of 5 mg/kg and 10 mg/kg with CPX at 10 mg/kg, administered 4 h post-infection, demonstrated significant efficacy, resulting in a reduction of approximately 1.86 and 2.34 log_10_, respectively, compared to the untreated control after 24 h. CPX (50 mg/kg) showed a reduction of 1.9 log_10_ that is approximately equal to that of CPX (10 mg/kg) + **PQK4F** (10 mg/kg). Notably, the combination of CPX with **PQQ16P** shows higher CFU reduction than CPX alone at 50 mg/kg.

## 3. Discussion

*S. aureus* exhibits remarkable adaptability to environmental changes and rapidly acquires resistance to antibiotics. This study focuses on a key mechanism that contributes to heightened resistance against various antimicrobial agents, namely the expulsion of antibiotics through efflux pumps. Consequently, compounds capable of inhibiting these pumps may prove effective in countering or mitigating resistance. Therefore, the objective of our investigation was to conduct a thorough investigation of the impact of **PQQ16P** and **PQK4F** (Figure 1), two previously reported *S. aureus* NorA EPIs [42,58], on fluoroquinolone resistance or tolerance in *S. aureus*. The selection of these two compounds was based on their proven efficacy as NorA inhibitors, resulting from a series of medicinal chemistry optimization cycles. Indeed, both compounds have been demonstrated to exhibit synergistic activity with CPX at low concentrations against the *S. aureus* SA-1199B strain. Moreover, their activity is comparable to that of some of the most potent analogues reported in the literature as NorA EPIs [34,39,57]. In addition, recent efforts have been made to elucidate the mechanisms by which **PQQ16P** interacts with NorA through the use of supervised molecular dynamics (SuMD) and molecular docking simulations [66].

Thus, herein, we initially confirmed the ability of **PQK4F** and **PQQ16P** to synergize with CPX against SA-1199B (Table S1). We then proceeded to ascertain that the observed effect was primarily due to the actual inhibition of the NorA efflux pump. The initial indication of this phenomenon was observed in the absence of a synergistic effect between the two compounds and CPX when tested against *S. aureus* K1758, which lacks the *norA* gene (Table S2). Indeed, it is to be expected that compounds which act as NorA inhibitors will be unable to synergize with CPX against a strain which does not possess the NorA pump. Conversely, compounds that exhibit synergistic activity with CPX, even in strains lacking NorA, could potentially function as inhibitors of other efflux pumps that are otherwise basally expressed in this strain or act through non-specific mechanisms, such as alteration of the bacterial membrane. Therefore, to eliminate the potential influence of **PQK4F** and **PQQ16P** on the bacterial membrane, we also assessed their impact on membrane depolarization, membrane permeability, ATP depletion (Figure 3A–C), and their potential to counteract the activation of eukaryotic Ca^2+^ channels (Figure 4). Indeed, also the inhibition of the eukaryotic Ca^2+^ channels can contribute to identification of false EPIs, as demonstrated by compounds like verapamil [61,62]. It is noteworthy that both compounds did not exhibit the aforementioned undesirable activities, with the exception of membrane depolarization at the highest concentrations tested. In contrast, at lower concentrations that are useful for obtaining the synergistic effect with CPX, membrane depolarization is either modest or absent. This indicates that both compounds can act as direct NorA inhibitors devoid of a “dirty” effect. Once confirmed that the synergistic effect of **PQK4F** and **PQQ16P** was based on the inhibition of NorA, additional studies have been conducted to investigate the potential biological effect of combining NorA inhibitors with CPX against the resistant *S. aureus* strain. Time–kill curves demonstrated that the combination of CPX at concentrations of 2 µg/mL (one-quarter MIC) and 1 µg/mL (one-eighth MIC) with **PQK4F** at 12.48 µg/mL and **PQQ16P** at 6.24 µg/mL resulted in a notable reduction in *S. aureus* growth after 12 h of treatment, in comparison to the treatment involving CPX alone (Figure 2). It is worth noting that the concentrations of **PQK4F** and **PQQ16P** used in this study did not elicit significant depolarizing activity, as evidenced by membrane depolarization assays (Figure 3B). This suggests that the observed bacterial growth delay in time–kill curves for combinations of EPIs and CPX may be attributable to NorA inhibition. This finding highlights the potential of this adjunctive therapy to enhance the bactericidal effects of CPX, even at sub-MIC concentrations, against the target pathogens. Furthermore, both **PQK4F** and **PQQ16P** facilitated the enhancement of PAE, impeding bacterial cell recovery. At a concentration of 12.48 µg/mL, **PQK4F** and **PQQ16P** augmented the PAE of CPX at 2 µg/mL (one-quarter MIC) significantly, extending it by 3.7 h and 5.2 h, respectively, in comparison to CPX at its MIC (8 µg/mL), which exhibited a PAE of 1.3 h. In addition to enhancing the efficacy of antibiotic treatment in suppressing the growth of *S. aureus*, both compounds demonstrated a notable reduction (8-fold) in the MPC of CPX when administered in combination, as evidenced by mutation frequency analysis (Table 1). These findings highlight the potential of a concurrent therapy in minimizing the development of antibiotic resistance. Indeed, the enhancement of efflux pump expression in bacteria represents a crucial initial step in the development of target-based resistance [17,67]. Considering these findings, the combination of EPIs and antibacterials may represent a promising avenue for future therapeutic development. This approach has the potential to prevent the emergence of resistance, even when utilizing lower concentrations of antibacterials. Furthermore, the inhibition of the NorA efflux pump may prove an effective method of reducing the capacity of *S. aureus* to invade macrophages (Figure 9). Although *S. aureus* is typically not considered an intracellular pathogen, there is a possibility for it to survive and propagate disease by briefly occupying an intracellular niche [65,68]. It is worth noting that our observations align with previous research [51] and provide evidence that overexpression of the NorA efflux pump contributes to the intracellular invasion of *S. aureus* SA-1199B in THP-1 macrophage cell lines. This significantly enhances the invasion ability of SA-1199B compared to the wild-type strain SA-1199 and the *norA*-deleted strain SA-K1758. In addition, the NorA EPI compounds **PQK4F** and **PQQ16P** were observed to prevent invasion by SA-1199B, thereby further confirming their ability to inhibit NorA and corroborating the evidence that NorA plays a role in aiding *S. aureus* in invading macrophages.

To ascertain the potential utility of these two compounds in an animal model, the toxicity profile was also investigated using three distinct mammalian cell lines (RAW, HEK, and HepG2 cell lines—Figure 5). Both compounds demonstrated only modest toxicity at the highest concentrations tested, which are considerably higher than those required to achieve the potent synergistic effect with CPX. Furthermore, when evaluated via comet assays at concentrations up to 12.5 µg/mL on the same three cell lines, neither compound exhibited any notable genotoxicity (Figure 7). Moreover, both compounds demonstrated minimal hemolytic activity, with a rate of less than 3% (Figure 6).

Subsequently, encouraged by these promising results, we decided to assess the in vivo effectiveness of the combination of **PQK4F** and **PQQ16P** with CPX. It is crucial to emphasize the significance of these studies in animal models, as they facilitate the assessment of the actual potential of the NorA EPI–CPX combination. Indeed, while numerous examples of NorA EPIs exist in the literature, regrettably, there is a dearth of data pertaining to their potential applicability in animal models, which would facilitate more comprehensive preclinical studies. In this study, our findings revealed a significant reduction in CFU of **PQK4F** or **PQQ16P** in combination with CPX, both compared to infected animals treated with CPX alone and the control group of untreated animals. Indeed, the combination of NorA EPIs with 10 mg/kg of CPX showed an activity comparable or slightly better (in the case of **PQQ16P**) than CPX alone at 50 mg/kg, strongly highlighting the advantage of combining NorA EPIs with CPX in animal models.

## 4. Materials and Methods

### 4.1. Bacterial Strains, Cell Lines, and Growth Medium

The *S. aureus* SA-1199B (overexpressing the NorA MDR efflux pump and bearing an A116E GrlA substitution), SA-1199 (*norA* wild-type), and SA-K-1758 (NCTC 8325-4 *norA* deletion mutant) were used. Bacterial media included cation-adjusted BBL^TM^ Mueller Hinton II Broth (CA-MHB), and Mueller–Hinton agar (MHA).

### 4.2. MIC Determination and Synergy Assay

Using broth microdilution by CLSI standards, the minimum inhibitory concentration (MIC) was ascertained [69,70]. The experiment used 96 round-bottom well plates with a total reaction volume of 200 µL. The bacteria were diluted until each well contained 5 × 10^5^ CFU/mL and their OD_600nm_ was set to 0.25–0.3. The plates were then incubated at 37 °C for 18 h.

The checkerboard experiment was used to determine the synergistic effect of the compounds **PQK4F** and **PQQ16P** with CPX (Sigma Aldrich Chemical Co., St. Louis, MO, United States) [69,70]. The fractional inhibitory concentration index (FICI) was employed to evaluate the synergistic effect of the compounds **PQK4F** and **PQQ16P**. The FICI was calculated using the following formula:

FICI = FIC [A] + FIC [B];

FIC [A] = [MIC of antibiotic in the presence of EPI/MIC of antibiotic alone];

FIC [B] = [MIC of EPI in the presence of antibiotic/MIC of EPI alone].

For a combination to be synergistic, its FICI value should be ≤0.5, and if the FICI is between 0.5 and 4, it is considered indifferent, and if the FICI value is >4, it is considered antagonistic [71].

### 4.3. Membrane Potentiation Assay

Mid-exponential *S. aureus* SA-1199B was cultivated at 37 °C. The cells were centrifuged, washed three times for 10 min at 4000× *g*, and then resuspended in 50 mM HEPES buffer with 0.1% glucose. Finally, the OD_600nm_ was adjusted to 0.1. The cells were then treated with DiSC_3_(5) (Sigma Aldrich) dye at a concentration of 1 µM and 300 mM KCl in HEPES buffer (to balance the outer and cytoplasmic K^+^ concentrations) before being incubated for 10 min at room temperature in the dark [72,73,74,75]. Valinomycin (Sigma Aldrich) (MIC = 16 µg/mL) was used as the positive control and the compounds **PQK4F** and **PQQ16P** were added at 0.78, 1.56, 3.12, 6.24, and 12.48 µg/mL concentrations. After that, the bacterial OD_600nm_ was normalized for both the control and all treatment groups. Then, the fluorescence leakage was measured after 30 min at a λ_excitation_/λ_emission_ of 622/670 nm.

### 4.4. Membrane Permeability Assay

The Invitrogen^TM^ Propidium Iodide (PI) kit (Thermo Fisher Scientific, Waltham, MA USA) methodology was modified slightly for the membrane permeabilization assay to quantify the fluorescence of propidium iodide (PI) permeating the membrane. *S. aureus* SA-1199B was cultured and allowed for growth in CA-MHB until it reached the mid-exponential stage with an OD_600_ nm of 0.3. The compounds **PQK4F** and **PQQ16P** at 6.24 µg/mL and 12.48 µg/mL concentrations were added to the 500 µL bacterial suspension for 30 min. Following treatment, the culture was washed three times and resuspended in PBS. The OD_600nm_ was normalized for treated and untreated cells. The suspensions were given a 30 µM PI treatment and incubated for 15–20 min at room temperature. The suspensions were then suspended in 500 µL of PBS after being centrifuged at 13,000× *g* for 5 min to remove any unbound PI. After 30 min, the fluorescence of 100 µL of suspensions was measured in a 96-well plate at 490 and 635 nm for excitation and emission, respectively [39].

### 4.5. Determination of Intracellular ATP Levels

Bacteria’s intracellular ATP levels were measured by the manufacturer’s instructions using an ATP determination kit (Invitrogen, Life Technologies, USA). An amount of 500 µL of the *S. aureus* SA-1199B bacterial culture was treated with CCCP (Sigma Aldrich) at 6.24, 12.48, 24.96 µg/mL in MHB and the compounds **PQK4F** and **PQQ16P** separately at a sub-inhibitory concentration for 4 h at 37 °C and 200 rpm. After treatment, the OD_600nm_ of the treated and untreated culture was normalized. An amount of 100 µL of freshly prepared solution of BacTiter-Glo reagent were added in each well and then an equal volume of cells was added and incubated for 5 min. All steps were performed at room temperature (22–25 °C). Total ATP was measured using relative luminescence [39,76]. An 0.25–1 s integration time per well should be a guideline.

### 4.6. Mammalian Ca^2+^ Channel Blocking Assay

The Fluo-4 Direct Ca^2+^ channel test kit (Life Technologies, Carlsbad, CA, USA) was utilized to investigate the effect of the compounds **PQK4F** and **PQQ16P** on human calcium channels by the manufacturer’s recommendations [62]. Fluorescence was evaluated when Probenecid (5 mM) and Fluo-4 dye were added to HEK 293T cells (5 × 10^4^) for 1 h. The compounds **PQK4F** and **PQQ16P** at 6.24 µg/mL and 12.48 µg/mL concentrations, and verapamil at 50 μg/mL were then applied to the cells. After 2 min, the Ca^2+^ channel stimulator carbachol (50 μg/mL) was applied, and the fluorescence was measured at 494/516 nm using a microplate reader.

### 4.7. Genotoxicity Test

#### 4.7.1. Chemicals and Reagents

Minimum Essential Medium Eagle, Dulbecco′s Modified Eagle′s Medium, fetal bovine serum (FBS), trypsin-EDTA, L-glutamine, antibiotics (penicillin and streptomycin), non-essential amino acid, β-mercaptoethanol, and HEPES were bought from Euroclone SpA (Milan, Italy). Hydrochloric acid (HCl), dimethyl sulfoxide (DMSO), ethanol, ethylenediaminetetraacetic acid disodium (Na_2_EDTA) and tetrasodium (Na_4_EDTA) salt, sodium chloride (NaCl), and sodium hydroxide (NaOH) were obtained from Carlo Erba Reagents Srl (Milan, Italy). Dulbecco’s phosphate-buffered saline, pH 7.4 (PBS), ethidium bromide, low- and normal-melting point agarose (LMPA and NMPA, respectively), 4-nitroquinoline *N*-oxide (4NQO), tris(hydroxymethyl)aminomethane (Tris base), Triton X-100, and GlutaMAX were obtained from Merck Life Science (Merck KGaA, Darmstadt, Germany). Conventional microscope slides and coverslips were purchased from Knittel-Glaser GmbH (Braunschweig, Germany). Distilled water was used throughout the experiments.

#### 4.7.2. Alkaline Single-Cell Microgel Electrophoresis (Comet) Assay

According to the Organization for Economic Cooperation and Development (OECD) guidelines [77,78], to avoid any conditions which would lead to false-positive results, only non-cytotoxic concentrations (i.e., 3.125, 6.25, 12.5 µg/mL) of **PQK4F** or **PQQ16P** were considered in the comet assay. This latter was performed following previous procedures [78,79], with minor modifications.

Firstly, each cell line (HEK 293T from passage 2 to 4, RAW 264.7 from passage 2 to 4 and HepG2 from passage 7 to 10) was seeded at approximately 2.65 × 10^5^ cells/well in 12-well plates (Corning Inc, Corning, NY, USA). The day after, the growth medium was removed and cells were exposed to the selected non-cytotoxic scalar concentration (see above). Proper negative (untreated cells) and positive (2 µM NQO) controls were carried out. In order to prevent any additional DNA damage, each step was carried out under a yellow light throughout the comet test, which was set up in triplicate.

After 4 h of treatment, cells were detached and centrifuged (1000× *g*, 8 min, 8 °C). Subsequently, they were resuspended in 0.7% LMPA (in Ca^2+^/Mg^2+^-free PBS (*w*/*v*)) and stratified onto microscope slides precoated with 1% NMPA in Ca^2+^/Mg^2+^-free PBS (*w*/*v*). Samples were put in the fridge (4 °C) with proper coverslips, allowing the agarose solidification. After 10 min, another top layer of 75 µL of 0.7% LMPA was added. Slides were immersed overnight at 4 °C in a lysing solution (2.5 M NaCl, 100 mM Na_2_EDTA, 10 mM Tris-HCl, pH 10, and 1% Triton X-100).

The day after, samples were kept for 40 min in an alkaline buffer solution (pH > 13) of 10 mM Na_4_EDTA and 300 mM NaOH, promoting both alkali-labile damage expression and the unwinding of DNA. Subsequently, electrophoresis was performed for 20 min with a horizontal box (HE99; Hoefer Scientific, Holliston, MA, USA) placed in an ice bath. The electric field strength was set at 1 V/cm and 300 mA of current (Power Supply E411; Consort, Turnhout, Belgium). Microgels were later subjected to a neutralized buffer (0.4 M Tris-HCl buffer, pH 7.5).

Finally, slides were fixed in 70% ethanol and absolute ethanol for 15 min plus 15 min, respectively. Samples were stored at room temperature.

To measure the DNA damage, percentages of tail intensity of one hundred arbitrary cells (50 cells each replicate slide) were examined using an Olympus BΧ41 (Olympus Corporation, Tokyo, Japan) fluorescence microscope equipped with a high-sensitivity charge-coupled device (CCD) camera connected to image analysis system (“Comet Assay III”, Perceptive Instruments Ltd, Haverhill, UK), after staining the slides with ethidium bromide (50 µL, 20 µg/mL). The median of tail intensity percentages of scored comets was used to calculate the group means [80].

Results were expressed as the ratio of means (treated cells % tail intensity/untreated cells % tail intensity), for each concentration. Results were expressed as the ratio of means with a 95% confidence interval of the triplicate test. Statistical significance was rated through one-way ANOVA and Dunnet’s post hoc test considering a significant level of *p* < 0.05.

### 4.8. Post-Antibiotic Assay

The *S. aureus* SA-1199B strain was cultivated to the mid-log phase in CA-MHB. After that, the culture was set up to an OD_600nm_ of 0.25. The bacterial culture was further diluted a thousand times before being treated with CPX at concentrations of 8 and 2 µg/mL, and the compounds **PQK4F** and **PQQ16P** at 3.12, 6.24, 12.48 µg/mL concentrations or a combination of CPX at 2 µg/mL with **PQK4F** or **PQQ16P** at 3.12, 6.24, 12.48 µg/mL concentrations, at 37 °C for two h. Following that, the bacterial suspension OD_600nm_ for each treatment group and the control was normalized. Then, to remove the drug’s carry-over effect, the bacterial cultures were centrifuged for 10 min at 1200× *g*. The control was the untreated bacterial suspension. In a 96-well flat-bottom plate, a 300 µL bacterial suspension was dispensed and subjected to incubation at 37 °C for 18 h. The Post-Antibiotic Effect (PAE) was determined using the formula PAE = T_50_ − C_50_, where T_50_ and C_50_ represent the durations required for the treated and untreated bacterial cultures, respectively, to achieve 50% of the optical density (OD) observed in the untreated culture [81].

### 4.9. Mutation Prevention Concentration (MPC)

Bacterial suspensions of *S. aureus* SA-1199B (10^9^ CFU) were applied to MHA plates at different CPX concentrations (0.25×, 0.5×, 1×, 2×, 4×, 8 × MIC) alone and in combination with **PQK4F** at 6.24 and 12.48 µg/mL and **PQQ16P** at 3.12 and 6.24 µg/mL concentrations, and the plates were then incubated at 37 °C. Colonies were counted after 48 h, and the mutation frequency was calculated by dividing the number of survivors by the total CFU plated. The concentration at which no colonies formed was referred to as the MPC [82].

### 4.10. Time–Kill Kinetics Study

*S. aureus* SA-1199B cultures were cultivated to mid-exponential phase, and a suspension with an optical density at 600 nm (OD_600nm_) of approximately 0.3 was prepared. The initial inoculum of *S. aureus* SA-1199B was exposed to CPX at concentrations of 8 µg/mL and 2 µg/mL and **PQK4F** and **PQQ16P** at its sub-inhibitory concentrations of 12.48 µg/mL and 6.24 µg/mL, respectively, and also, a combination of CPX with **PQK4F** and **PQQ16P** separately at 12.48 µg/mL and 6.24 µg/mL, respectively. Bacterial counts were determined at various intervals (0, 2, 4, 6, 8, 12, and 24 h) and quantified as colony-forming units per milliliter (CFU/mL) [76].

### 4.11. Hemolysis Assay

For the hemolysis assay [76], fresh rabbit blood erythrocytes were employed. Blood was drawn into a heparin tube, then centrifuged and washed with PBS three times. The RBCs were resuspended in PBS to achieve a final concentration of 4% (*v*/*v*). In 200 µL of freshly prepared 4% RBC suspension, **PQK4F** and **PQQ16P** were added at final concentrations ranging from 0.78 to 100 µg/mL. Additionally, 0.1% Triton-X (positive control) was included. The suspension was then incubated for 1 h at 37 °C before being centrifuged for 5 min at 4 °C at 1000× *g*. Following centrifugation, 100 µL of the supernatant was transferred to a 96-well flat-bottom plate, and the absorbance was measured at 570 nm using a BioTek spectrophotometer (Highland, VT, USA). % Hemolysis = [(Absorbance of treatment − Absorbance of blank)/(Absorbance of control − Absorbance of blank)] × 100.

### 4.12. Mammalian Cytotoxicity

The cytotoxicity of compounds **PQK4F** and **PQQ16P** was evaluated on Human Embryonic Kidney 293T cells (HEK), RAW cells, and HepG2 cells [39,76]. Each well was seeded with approximately 10,000 cells separately in individual plates and then incubated in a CO_2_ incubator with 5% CO_2_ at 37 °C for 24 h. Following incubation, varying concentrations of compounds **PQK4F** and **PQQ16P** (1.56, 3.125, 6.25, 12.5, 25, 50, 100 µg/mL) were added to the respective plates and further incubated at 37 °C in a CO_2_ incubator. After further 24 h of incubation, MTT solution (500 µg/mL) was added to the wells after removing the medium. Formazan crystals formed were then solubilized with a 100 µL stopping solution comprising 40% (*v*/*v*) dimethylformamide (DMF) in 2% (*v*/*v*) glacial acetic acid and 16% (*w*/*v*) sodium dodecyl sulfate (SDS) at pH 4.7. The absorbance of the resulting solution was measured at 570 nm using a BioTek spectrophotometer.

The following equation was used to calculate the percentage viability:

% Cell viability = (A_t_/A_c_) × 100;

A_t_ = (absorbance of test compound − absorbance of blank);

A_c_ = (absorbance of control − absorbance of blank).

### 4.13. Macrophage Invasion Assay

The THP-1 macrophage cell line was utilized to investigate the intracellular invasion of *S. aureus* in the presence of compounds **PQK4F** and **PQQ16P** [39,76]. *S. aureus* strains SA-1199B, SA-1199, and K-1758 (10^6^ CFU/well) were used to infect macrophages (10^5^ cells/well), with or without **PQK4F** and **PQQ16P** (12.48 and 6.24 µg/mL), and incubated for 2 h. Subsequently, extracellular bacteria were removed by washing the cells three times with PBS, followed by treatment with gentamicin (50 µg/mL) for 30 min. The cells were then briefly treated with 0.1% saponin, and the viability of intracellular bacteria was determined by plating on MHA.

### 4.14. In Vivo Thigh Infection Model

Eight groups of mice, each consisting of six female BALB/c mice, were employed for the study. To induce neutropenia, female mice were treated with two intraperitoneal doses of 150 mg/kg and 100 mg/kg of cyclophosphamide, administered 4 and 1 days prior to infection, respectively. Following this, mice were intramuscularly injected with 50 µL of *S. aureus* SA-1199B (10^7^ CFU/mL). Four h post-infection, various treatments were administered subcutaneously. Two groups received 5 mg/kg and 10 mg/kg of either **PQK4F** or **PQQ16P** individually. Another set of two groups received a combination of 5 mg/kg and 10 mg/kg of **PQK4F** or **PQQ16P** with 10 mg/kg of CPX (Sigma Aldrich). Subsequently, two groups were administered with 10 mg/kg and 50 mg/kg of CPX, respectively. The final two groups served as untreated controls. One control group was euthanized before treatment by cervical dislocation after 4 h of infection. The right thighs were then excised, homogenized, and CFUs were determined. The remaining groups were euthanized by cervical dislocation 20 h after receiving the administered doses. CFUs from all groups were enumerated from the right thigh [39,83].

### 4.15. Statistical Analysis

Data are presented as mean ± standard deviation. Two-tailed *t*-tests were conducted to detect group variances. Statistical significance was defined as *p* < 0.05 (*), highly significant as *p* < 0.01 (**), *p* < 0.001 (***), and *p* < 0.0001 (****).

## 5. Conclusions

In this study, a comprehensive analysis was conducted to elucidate the mechanism of action of two NorA EPIs that had been previously identified. Subsequently, an investigation was conducted to assess the potential benefits of combining both EPIs with CPX, with the aim of identifying the potential advantages of this combination. In conclusion, our findings demonstrate that the NorA EPI-CPX combination is advantageous in several aspects. These include increasing the efficacy and potency of CPX against resistant strains of *S. aureus*, as well as preventing the ability of *S. aureus* to generate mutations and become resistant. Moreover, NorA EPIs can also exert an anti-virulence action, limiting the invasion of macrophages by *S. aureus*. It is also noteworthy that the combination of NorA EPIs with CPX has been demonstrated to be effective in the treatment of *S. aureus* infection in an animal model. It is crucial to acknowledge that this animal model study employed the resistant strain SA-1199B, which exhibits both overexpression of the NorA efflux pump and a single mutation on the fluoroquinolone target. Nevertheless, it is essential to underscore the notion of combining a NorA EPI with CPX also to prevent the emergence of resistance. It is therefore our recommendation that the concept of exclusively utilizing EPIs to treat infections with pre-existing resistance be reconsidered. Conversely, it would be prudent to endeavor to demonstrate that the EPI-antibacterial combination has the potential to serve as a significant future strategy for the prevention of the emergence of resistant strains. The efficacy of this approach has been validated in this study through in vitro MPC experiments. Further studies will be needed to elucidate the potential advantages of this combination as a prophylactic strategy against resistance development in animal models.

## Figures and Tables

**Table 1:** Mutation prevention concentration of CPX alone at various concentrations (0.25 to 8 × MIC) and in combination with **PQQ16P** at 6.24 and 12.48 µg/mL and **PQK4F** at 3.12 and 6.24 µg/mL on *S. aureus* SA-1199B.

CPX Conc.	0.25 × MIC	0.5 × MIC	1 × MIC	2 × MIC	4 × MIC	8 × MIC
	**CFU Count**					
CPX alone	UC	UC	UC	2.75 × 10^−9^	2.3 × 10^−8^	<10^−9^
+**PQK4F** (6.24 µg/mL)	UC	1 × 10^−9^	<10^−9^	<10^−9^	<10^−9^	<10^−9^
+**PQK4F** (12.48 µg/mL)	5.26 × 10^−9^	3 × 10^−7^	<10^−9^	<10^−9^	<10^−9^	<10^−9^
+**PQQ16P** (3.12 µg/mL)	UC	1.67 × 10^−9^	<10^−9^	<10^−9^	<10^−9^	<10^−9^
+**PQQ16P** (6.24 µg/mL)	UC	1 × 10^−7^	<10^−9^	<10^−9^	<10^−9^	<10^−9^

UC = Uncountable; CPX (MIC) = 8 µg/mL.

## Data Availability

The original contributions presented in this study are included in the article/Supplementary Materials. Further inquiries can be directed to the corresponding authors.

## Supplementary Materials

The following supporting information can be downloaded at: https://www.mdpi.com/article/10.3390/antibiotics14040339/s1, Table S1: MIC and checkerboard synergy assay of the compounds **PQK4F** and **PQQ16P** with CPX on *S. aureus* SA-1199B (*norA* overexpressed strain); Table S2: MIC and checkerboard synergy assay of the compounds **PQK4F** and **PQQ16P** with CPX on *S. aureus* K-1758 (*norA* deletion strain); Table S3: Genotoxicity assessment of **PQK4F** and **PQQ16P** at different concentrations.

## Abbreviations

AMR: antimicrobial resistance; ARBs: antimicrobial resistance breakers; CPX: ciprofloxacin; EPI: efflux pump inhibitor; EtBr: ethidium bromide; FICI: fractional inhibitory concentration index; HEK 293T: human embryonic kidney cells; HepG2: hepatoblastoma cell line; MDR: multidrug-resistance; MFS: Major Facilitator Superfamily; MIC: minimum inhibitory concentration; MPC: mutation prevention concentration; PAE: post-antibiotic effect; MRSA: methicillin-resistant *S. aureus*; PMF: proton motive force.
