# Supplementary material for: In Vitro and In Vivo Investigations into the Potential of Quinazoline and Quinoline Derivatives as NorA Efflux Pump Inhibitors Against Resistant Staphylococcus aureus Strains
Source: Antibiotics (Basel). 2025 Mar 26;14(4):339. doi: 10.3390/antibiotics14040339 (PMC12024145; doi:10.3390/antibiotics14040339)
Supplement: Supplementary file 1 [file antibiotics-14-00339-s001.zip › antibiotics-3504133-supplementary.pdf]

In vivo and in vitro Investigations into the Potential of Quinazoline and Quinoline Derivatives as NorA Efflux Pump Inhibitors against Resistant *Staphylococcus aureus* Strains

Nishtha Chandal[1,2,3], Midhi Sharma[1,2], Giada Cernicchi[4], Tommaso Felicetti[4], Tommaso Rondini[5], Mattia Acito[5,6], Hemraj Nandanwar[1,2,*], and Stefano Sabatini[4,*]

[1]*Clinical Microbiology & Antimicrobial Research Laboratory, CSIR-Institute of Microbial Technology, Sector 39-A, Chandigarh-160036, India*

[2]*Academy of Scientific & Innovative Research (AcSIR), Ghaziabad, Uttar Pradesh-201002, India*

[3]*Institute of Biosciences and Technology, Texas A & M Health Science Centre, Houston, USA (present address)*

[4]*Department of Pharmaceutical Sciences, Section of Chemistry and Drug Technology, University of Perugia, Perugia-06123, Italy*

[5]*Department of Pharmaceutical Sciences, Section of Biochemical and Health Sciences, University of Perugia, Perugia-06123, Italy*

[6]*School of Medicinal and Health Products Sciences, University of Camerino, Camerino, Italy (present address)*

Table of Contents

Table S1. MIC and checkerboard synergy assay of the compounds **PQK4F** and **PQQ16P** with CPX on *S. aureus* SA-1199B (*norA* overexpressed strain) S2

Table S2. MIC and checkerboard synergy assay of the compounds **PQK4F** and **PQQ16P** with CPX on *S. aureus* K-1758 (*norA* deletion strain) S3

Table S3. Genotoxicity assessment of **PQK4F** and **PQQ16P** at different concentrations S4

Table S1: MIC and checkerboard synergy assay of the compounds **PQK4F** and **PQQ16P** with CPX on *S. aureus* SA-1199B (*norA* overexpressed strain)

**Compd.**	EPI MIC (µg/mL)	EPI Conc.	CPX		
			MIC (µg/mL)	Modulation Factor	FICI
**PQK4F**	>400	12.48	1	8	0.156
		6.24	2	4	0.265
		3.12	2	4	0.257
		1.56	4	2	0.503
		0.78	4	2	0.501
		0	8	-	1
**PQQ16P**	≥100	12.48	0.5	16	0.187
		6.24	1	8	0.187
		3.12	2	4	0.281
		1.56	4	2	0.515
		0.78	4	2	0.507
		0	8	-	1
**Reserpine**	128	32	0.5	16	0.312
		16	1	8	0.25
		8	2	4	0.312
		4	4	2	0.531
		2	8	-	1.015
		-	8	-	1

Table S2: MIC and checkerboard synergistic assay of the compounds **PQK4F** and **PQQ16P** with CPX on *S. aureus* K-1758 (*norA* deletion strain)

**Compd.**	EPI MIC (µg/mL)	Conc.	CPX		
			MIC (µg/mL)	Modulation Factor	FICI
**PQK4F**	64	12.48	0.0625	2	0.89
		6.24	0.0625	2	0.695
		3.12	0.125	-	1
		1.56	0.125	-	1
		0.78	0.125	-	1
		0	0.125	-	1
**PQQ16P**	32	12.48	0.0625	2	0.89
		6.24	0.0625	2	0.695
		3.12	0.125	-	1
		1.56	0.125	-	1
		0.78	0.125	-	1
		0	0.125	-	1
**Reserpine**	128	32	0.0312	4	0.5
		16	0.125	-	1
		8	0.125	-	1
		4	0.125	-	1
		2	0.125	-	1
		-	0.125	-	1

Table S3. Genotoxicity assessment of **PQK4F** and **PQQ16P** at different concentrations. Results were expressed as the ratio of means with 95% CI.

**HEK 293T cell line**		
**Samples**	**Ratio of means**	**95% CI**
Negative Control	1.00	
PQK4F 3.125 µg/mL	1.18	0.85 – 1.64
PQK4F 6.25 µg/mL	1.37	0.99 – 1.91
PQK4F 12.5 µg/mL	1.09	0.73 – 1.63
4NQO (2 µM)	5.00	3.54 – 7.05
**Samples**	**Ratio of means**	**95% CI**
Negative Control	1.00	
PQQ16P 3.125 µg/mL	1.29	0.97 – 1.73
PQQ16P 6.25 µg/mL	1.30	0.95 – 1.77
PQQ16P 12.5 µg/mL	1.11	0.83 – 1.47
4NQO (2 µM)	5.45	4.16 - 7.13
**RAW 264.7 cell line**		
**Samples**	**Ratio of means**	**95% CI**
Negative Control	1.00	
PQK4F 3.125 µg/mL	1.17	0.94 – 1.45
PQK4F 6.25 µg/mL	1.02	0.76 - 1.38
PQK4F 12.5 µg/mL	1.18	0.85 – 1.64
4NQO (2 µM)	4.24	3.35 – 5.38
**Samples**	**Ratio of means**	**95% CI**
Negative Control	1.00	
PQQ16P 3.125 µg/mL	1.01	0.92 – 1.12
PQQ16P 6.25 µg/mL	0.79	0.71 – 0.90
PQQ16P 12.5 µg/mL	0.96	0.77 – 1.20
4NQO (2 µM)	3.23	2.75 – 3.79
**Hep G2 cell line**		
**Samples**	**Ratio of means**	**95% CI**
Negative Control	1.00	
PQK4F 3.125 µg/mL	1.16	0.86 – 1.56
PQK4F 6.25 µg/mL	1.16	0.87 – 1.55
PQK4F 12.5 µg/mL	1.14	0.90 – 1.46
4NQO (2 µM)	5.16	4.08 – 6.53
**Samples**	**Ratio of means**	**95% CI**
Negative Control	1.00	
PQQ16P 3.125 µg/mL	0.79	0.53 – 1.17
PQQ16P 6.25 µg/mL	0.78	0.63 – 0.98
PQQ16P 12.5 µg/mL	0.94	0.69 – 1.26
4NQO (2 µM)	3.86	3.48 – 4.30
